# Applying cultural psychiatry to non-pharmacological interventions

**DOI:** 10.1192/bjo.2026.12017

**Published:** 2026-07-07

**Authors:** Kamaldeep Bhui, Richard Porter, Katie Douglas, Javier I. Escobar, Steve Kisely, Arjune Sen, Kenneth R. Kaufman

**Affiliations:** Academic Department of Psychiatry, Wadham College, University of Oxford, Oxford, UK; Department of Psychological Medicine, University of Otago, Christchurch, New Zealand; Department of Psychiatry, Florida International University, Miami, USA; Department of Psychiatry, Rutgers University, New Brunswick, USA; Department of Psychiatry, University of Queensland, Brisbane, Australia; Oxford Epilepsy Research Group, Nuffield Department of Clinical Neuroscience, John Radcliffe Hospital/University of Oxford, Oxford, UK; Department of Psychiatry, Rutgers Robert Wood Johnson Medical School, New Brunswick, USA

**Keywords:** Transcultural psychiatry, adaptation, good practice, evidence-based mental health, psychological treatments

## Abstract

To mark the 10th Anniversary of *BJPsych Open*, we explore the contributions of papers published in *BJPsych Open* to advance cultural psychiatry practice and policy. In our overview of papers published in *BJPsych Open*, we found examples of good practice where authors detailed the translation methods and interpretation models in the research. The task facing clinicians and public health practitioners is to evolve applied, locally relevant, culturally competent interventions in which specific adaptations are shaped by the potential beneficiaries, alongside theoretical and practical issues of cultural adaptation. Researchers and clinicians will need to provide evidence of acceptability and effectiveness of adapted interventions, alongside considering financial and implementation realities.

**Figure:**
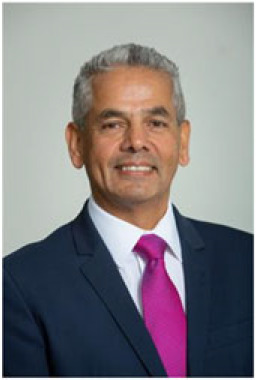

We are delighted to mark the 10th Anniversary of *BJPsych Open* by exploring the contributions of papers published in *BJPsych Open* to advance cultural psychiatry practice and policy. Cultural psychiatry seeks to understand the role of culture in the experience, expression and care of people living with mental illnesses.^1^ Culture is a complex variable that can be hard to define. Here, we consider that culture includes heritage, heritage-related identity, health beliefs, shared ideologies and practices related to morals, arts, laws and supernatural forces. Mental health professionals require practical and nuanced understanding of how culture influences the assessment and management of mental illnesses among people from diverse cultural groups. Cultural psychiatry research includes theoretical and practical advances to improve care for culturally diverse populations, informed by scholarship from sociology, anthropology, humanities, public health, political and linguistic sciences and philosophy.^2–4^

We examined all papers published in *BJPsych Open* and identified over 100 manuscripts that have relevance to cultural psychiatry. Most frequently, these manuscripts compared the rates of mental disorder across countries and ethnic/racial groups; assessed risk factors using validated item-based measures across countries and ethnic/racial groups; or examined treatment uptake and outcomes by country and ethnic/racial group. Differences by countries and/or racial–ethnic groups are often used as a proxy for the complex construct of culture as described earlier. Some articles proposed new systems of care in low- and middle- income countries (LMICs) and evaluated mental health policies, although not all studies explicitly addressed the cultural dimensions of psychiatry.

Only a minority of the papers focused on interventions and their effectiveness, which are arguably the most important, given their impact on patient care and policy. Therefore, in this Editorial, we showcase studies of non-pharmacological interventions published in *BJPsych Open*. Although this is not primary research or a systematic review, we considered papers that provided important information and held lessons for future research, policy and practice.

There are many elements of adapting interventions for LMICs that are relevant to all interventions. For example, given the shortage of trained practitioners, task-sharing encourages the deployment of local community members in other roles and jobs, and supports them in delivering mental healthcare.^5^ Similarly, reliance on local assets, including charities and faith communities, is often essential for acceptability and implementation.

Approaches to developing non-pharmacological psychiatric interventions in ethnically or racially minoritised populations attempt to adapt therapies that were originally developed in high-income countries (HICs), to align better with the local context and cultures. One possible negative aspect to this approach is that it could be seen as an extension of the imposition of health systems on previously colonised countries. More positively, these adaptations may be more likely to be widely accepted if there is already an evidence base, and interventions can be adapted and applied in an efficient and sustainable way. Adapting an intervention known to be effective in one country or cultural setting, and optimising its delivery in a new context, seems practical, feasible and less time consuming than developing an entirely new intervention for specific linguistic, cultural and ethnic groups.

We discuss specific instances in which non-pharmacological interventions, including psychotherapy and cognitive remediation, have been developed ‘*de novo*’ within the indigenous cultures of the population of interest, and compare these with the ostensibly ‘simpler’ process of adapting established effective therapies grounded in Western HICs. Both approaches may, of course, be necessary and iteratively used where adaptation or optimisation have failed. Some of the reasons for potential failures include diversity of health beliefs, variations in health literacy and potential acceptability of interventions. Any adapted intervention must also consider the cultural conceptualisation of the person, and personal agency and expectation of coping with misfortune and illness in culturally scripted ways.^6^

## Development and adaptation of therapies

An example of adaptation in an LMIC of a therapy developed in a Western culture is ‘culturally adapted’ psychoeducation for bipolar disorder (CaPE). The World Health Organization has specifically suggested that this mode of therapy for bipolar disorder be made available in all LMICs. CaPE has recently been examined both in Pakistan and in Nigeria.^7,8^ In both countries, the manual was adapted from the Barcelona Psychoeducation Programme for bipolar disorders.^9^

In Nigeria, a specific technique was used to adapt the Barcelona programme to deliver therapy in group sessions.^8^ The study adopted the Iterative Model of Co-adaptation to ensure that the CaPE manual and study protocol were culturally appropriate and suitable for Nigerian people with bipolar disorder. The method involved layers of consultation with people with bipolar disorder from the community for whom the intervention was being adapted. Despite small numbers (*N* = 34), the randomised controlled trial showed a significantly better improvement in depressive symptoms (as measured by the Patient Health Questionnaire-9) for CaPE plus treatment as usual (TAU) than for TAU alone. TAU included routine out-patient psychiatric care, psychotropic medications and other psychological treatments such as cognitive–behavioural or interpersonal psychotherapies for bipolar disorders, which are different from CaPE. Qualitative work suggested that CaPE was associated with an exploration of ‘culturally rooted interpretations of mental health, instilling confidence and a high sense of gratitude’. Some cultural beliefs that were discussed ascribed bipolar disorder to ‘spiritual causes that stem from the family of the affected patient’.

In contrast to these cultural adaptations of a Western therapy, Baheretibeb et al^10^ examined the feasibility of a collaboration between conventional, biomedically led psychiatry and traditional healers in Ethiopia. Neither traditional Ethiopian nor biomedical approaches were ‘adapted’, rather these were integrated into local systems of healing. In Ethiopia, a common treatment for mental illness involved the use of holy water, based on a belief that evil spirit possession is responsible for mental health issues and that holy water exorcises these demons. Psychiatrists and residents affiliated with Addis Ababa University established a collaboration with traditional healers, priests and patient attendants at two churches in Addis Ababa. There was a series of meetings, consultations and workshops. An important part of the process was the acknowledgement by both groups of previous mistreatment of patients.

In a retrospective, anonymous chart review of 1888 patients who attended the clinic between 2012 and 2019, most had severe mental illness, including schizophrenia (40%) and mood disorders (30%); 24% had substance misuse disorders. Nearly half (48%) of patients had some form of previous contact with biomedical treatment, but now presented for holy water treatment instead. The first contact for 88% of patients was from a non-biomedical source (faith-based prayer centres, general community traditional healers and holy water sites). Of those with previous biomedical treatment, 89% had discontinued their biomedical medication before presentation at the clinic. Following clinic attendance, 92% were comfortable in combining medication with simultaneous holy water treatment, although it was not feasible to formally track medication adherence, given the retrospective design. Adherence was enhanced by free provision of medication, psychoeducation, supervision by the attendants and endorsement by the priests. The study is an example of using the combination of culturally traditional and biomedical treatments in what seems to be a synergistic way. The lessons learned from respecting patient perspectives and facilitating dual treatment are for HICs as well. For example, if facilitating a complementary cultural therapy into a treatment plan also helps with medication adherence, positive patient outcomes could be amplified.

The assessment and treatment of cognitive disorders can present particular cross-cultural challenges. Tanveer and colleagues^11^ highlighted contrasting understandings of dementia in different culture. For example, increased tolerance of forgetfulness in the elderly in some cultures may militate against medical screening and treatment. An alternative approach to assessment in different cultures is therefore desirable.^12^

The increasing evidence for the use of cognitive remediation in schizophrenia (and in other mental health disorders) has led Press et al^13^ to adapt a commonly used computer-based cognitive rehabilitation programme (CIRCuiTS) for British South Asians. Adaptations included delivery in the primary language of the participant, engagement of family, inclusion of cultural requirements (e.g. *halal* items, as this was a Muslim cohort) in a shopping task, and that the treatment goals were discussed with the family at initial sessions. These cultural adaptations of CIRCuiTS were found to be acceptable, with high levels of engagement and satisfaction. Despite the small sample, the intervention led to improved cognition and mental state.

In a review of well-being and mental health interventions for indigenous people in prison, Perdacher et al^14^ identified five studies from the USA, Canada and Australia of interventions purely based on indigenous cultural practices rather than adapted from Western therapies. For example, Navajo sweat lodge ceremonies for alcohol use disorders involve a sauna-like experience, where physical suffering is combined with prayers and songs to the Creator. In a study of 190 participants, those who took part in more ceremonies had a greater reduction in alcohol consumption, although not significantly so.^15^ Other indigenous therapies such as *rongoā Māori* offer a holistic approach to health, including the use of native plant-based remedies, and physical therapies like massage (*mirimiri*) and spiritual healing in mainstream services in New Zealand, although standard randomised controlled trials to evaluate this approach have not been conducted.

## Recommendations for future research

In our overview of papers published in *BJPsych Open*, we found examples of good practice where authors detailed the translation methods and interpretation models in the research. Good practice should also include data-sharing, verification and interpretation with participants. These stages of research should be considered essential for inclusive research practice.^16^ Such aspects can be addressed during study design, and potentially improved by peer and editorial reviews. Recent papers have improved quality regarding these stages of research and heed these lessons.

In a detailed analysis of psychotherapy for Asian patients, Tseng^17^ proposed that adaptations of dominant Western origin psychotherapies need to consider philosophical, theoretical, practical and technical adjustments. Tseng’s nuanced consideration of adaptation research aligns with more recent global calls for scaling mental health interventions in LMICs and standardising reporting of cultural adaptations.^18,19^ Tseng distinguished the following approaches, which we commend for future research on non-pharmacological interventions in general:

- Culturally embedded or indigenous healing practices and their symbolic and metaphorical power.
- Culturally influenced unique therapies, evolved in a well-known paradigm within a particular cultural context, making it potentially suitable only for that cultural group.
- Cultural elements in mainstream therapies with minimal additions.
- Psychotherapy in different societies, where national and local preferences influence modalities and approaches.
- Intercultural therapies, wherein practitioners must be proficient in combining historical and sociocultural aspects into a new combined modality of therapy. It is apparent that many approaches developed within LMICs may be applicable in more resource privileged countries, particularly, but not exclusively, in marginalised communities. Cultural aspects apply across all peoples and all regions. Bidirectional, equitable and transparent learning will lead to greatest clinical advantage across settings.

With all of this in mind, the task facing clinicians and public health practitioners is to evolve applied, locally relevant, culturally competent interventions in which specific adaptations are shaped by the potential beneficiaries, as well as practical issues of cost, implementation and willingness of commissioners to pay. Policy and decision makers may prefer established effective interventions that can be efficiently optimised while remaining effective, given the harsh economic realities within healthcare in LMICs and HICs.

Hence, clinicians, researchers, policy makers and commissioners will need to align around the best evidence of acceptability, effectiveness, and financial and implementation realities with bidirectional learning between LMICs and HICs.

## References

[1] Bhui K , Gavrilovic JJ. 8 – Cultural psychiatry. In Core Psychiatry (eds P Wright , J Stern , M Phelan ): 105–13. W.B. Saunders, 2012.

[2] Kirmayer LJ. Cultural psychiatry in historical perspective. In Textbook of Cultural Psychiatry (eds D Bhugra , K Bhui ): 3–19. Cambridge University Press, 2007.

[3] Gómez-Carrillo A , Kirmayer LJ , Aggarwal NK , Bhui KS , Fung KP-L , Kohrt BA , et al. Integrating neuroscience in psychiatry: a cultural-ecosocial systemic approach. Lancet Psychiatry 2023; 10: 296–304. 36828009 10.1016/S2215-0366(23)00006-8

[4] Heller UC , Grant LH , Yasui M , Keysar B. Culturally anchored mental-health attitudes: the impact of language. Clin Psychol Sci 2024; 12: 290–304.

[5] Bhui K , Basu D , Nagpal S , Mutiso V , Pillai R , Hadfield K , et al. Acceptability and feasibility of a brief intervention to enhance resilience among young people and their families in India and Kenya. Glob Ment Health (Camb) 2024; 11: e86. 39464565 10.1017/gmh.2024.87 PMC11504944

[6] Kirmayer LJ. Psychotherapy and the cultural concept of the person. Transcult Psychiatry 2007; 44: 232–57. 17576727 10.1177/1363461506070794

[7] Husain MI , Chaudhry IB , Rahman RR , Hamirani MM , Mehmood N , Haddad PM , et al. Pilot study of a culturally adapted psychoeducation (CaPE) intervention for bipolar disorder in Pakistan. Int J Bipolar Disord 2017; 5: 3. 28155203 10.1186/s40345-017-0074-8 PMC5307423

[8] Jidong DE , Husain MI , Ike TJ , Khoso A , Taru MY , Nwoga CN , et al. A randomised controlled feasibility trial comparing culturally adapted psychoeducation and treatment as usual for persons with bipolar disorders in Nigeria. BJPsych Open 2025; 11: e133. 40574580 10.1192/bjo.2025.66 PMC12247066

[9] Stafford N , Colom F. Purpose and effectiveness of psychoeducation in patients with bipolar disorder in a bipolar clinic setting. Acta Psychiatr Scand Suppl 2013; 442: 11–8. 10.1111/acps.12118 23581788

[10] Baheretibeb Y , Wondimagegn D , Law S. Holy water and biomedicine: a descriptive study of active collaboration between religious traditional healers and biomedical psychiatry in Ethiopia. BJPsych Open 2021; 7: e92. 33947496 10.1192/bjo.2021.56 PMC8142542

[11] Tanveer S , Croucher MJ , Porter R. Cultural modification of neuropsychiatric assessment: complexities to consider. BJPsych Open 2022; 8: e68. 35287781 10.1192/bjo.2022.33 PMC8935941

[12] Dudley MD , Barker-Collo SL , Wilson DL , Garrett NK. Factors associated with Māori performance on the WAIS-IV. Arch Clin Neuropsychol 2019; 34: 1203–16. 30805644 10.1093/arclin/acy110

[13] Press C , Bamford J , Renwick L , Noke M , Drake R , Husain N. The feasibility of culturally adapted computerised cognitive remediation for first-episode psychosis. BJPsych Open 2025; 11: e52. 40099864 10.1192/bjo.2024.854 PMC12001949

[14] Perdacher E , Kavanagh D , Sheffield J. Well-being and mental health interventions for Indigenous people in prison: systematic review. BJPsych Open 2019; 5: e95. 31679537 10.1192/bjo.2019.80 PMC6854355

[15] Gossage JP , Barton L , Foster L , Etsitty L , LoneTree C , Leonard C , et al. Sweat lodge ceremonies for jail-based treatment. J Psychoactive Drugs 2003; 35: 33–42. 12733756 10.1080/02791072.2003.10399991

[16] Bhui K , Mooney R , Stepney M , Mand K , Kirk M , Lawrence E , et al. Improving research inclusion: learning from NIHR and Research Council funded studies in England. NIHR Open Res 2025; 5: 102.

[17] Tseng W-S. Culture and psychotherapy: Asian perspectives. J Ment Health 2004; 13: 151–61.

[18] Jordans MJD , Kohrt BA. Scaling up mental health care and psychosocial support in low-resource settings: a roadmap to impact. Epidemiol Psychiatr Sci 2020; 29: e189. 33239113 10.1017/S2045796020001018 PMC7737188

[19] Heim E , Weise C. Special issue editorial: cultural adaption of psychological interventions. Clin Psychol Eur 2021; 3: e7627. 36405679 10.32872/cpe.7627 PMC9670831

